# Identification and Bioactivity of Compounds from the Fungus *Penicillium* sp. CYE-87 Isolated from a Marine Tunicate

**DOI:** 10.3390/md13041698

**Published:** 2015-03-25

**Authors:** Lamiaa A. Shaala, Diaa T. A. Youssef

**Affiliations:** 1 Natural Products Unit, King Fahd Medical Research Center, King Abdulaziz University, Jeddah 21589, Saudi Arabia; E-Mail: lshalla@kau.edu.sa or lamiaelnady@yahoo.com; 2 Suez Canal University Hospital, Suez Canal University, Ismailia 51522, Egypt; 3 Department of Natural Products, Faculty of Pharmacy, King Abdulaziz University, Jeddah 21589, Saudi Arabia

**Keywords:** *Penicillium* sp., indole alkaloids, 1,4-diazepane derivatives, terretriones C and D, antimigration, antiproliferation, antimicrobial activity

## Abstract

In the course of our continuous interest in identifying bioactive compounds from marine microbes, we have investigated a tunicate-derived fungus, *Penicillium* sp. CYE-87. A new compound with the 1,4-diazepane skeleton, terretrione D (**2**), together with the known compounds, methyl-2-([2-(1H-indol-3-yl)ethyl]carbamoyl)acetate (**1**), tryptamine (**3**), indole-3-carbaldehyde (**4**), 3,6-diisobutylpyrazin-2(1H)-one (**5**) and terretrione C (**6**), were isolated from *Penicillium* sp. CYE-87. The structures of the isolated compounds were established by spectral analysis, including 1D (^1^H, ^13^C) and 2D (COSY, multiplicity edited-HSQC and HMBC) NMR and HRESIMS, as well as comparison of their NMR data with those in the literature. The compounds were evaluated for their antimigratory activity against the human breast cancer cell line (MDA-MB-231) and their antiproliferation activity against HeLa cells. Compounds **2** and **6** showed significant antimigratory activity against MDA-MB-231, as well as antifungal activity against *C. albicans*.

## 1. Introduction

**Figure 1:**
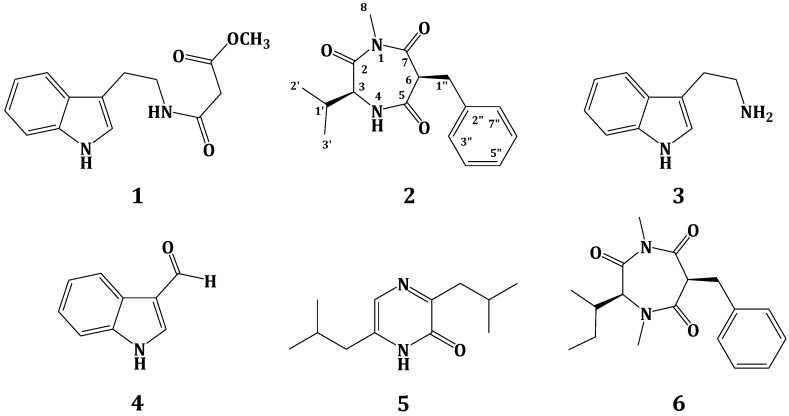
Structures of Compounds **1**–**6**.

Secondary metabolites from plants and microbes are considered as a vital component in drug discovery and development [1,2]. Marine-derived microbes, which have been neglected for a long time, have been isolated from different marine sources, including invertebrates, sediment and algae. These microbes were explored for the discovery of novel bioactive molecules [3,4]. Although members of the phylum Porifera (sponges) have received great attention from natural products chemists as producers of interesting bioactive compounds [5,6], they have become a privileged source of marine fungi [7,8,9,10,11,12,13,14] and their secondary metabolites with different bioactivities [15]. Marine fungi are known to be a rich source of bioactive compounds with potential biomedical and pharmaceutical applications [3,4]. The organic extract of the culture of a tunicate-derived fungus *Penicillium* sp. CYE-87 yielded a new compound, terretrione D (**2**), together with the known compounds, methyl-2-([2-(1H-indol-3-yl)ethyl]carbamoyl)acetate (**1**) [16], tryptamine (**3**), indole-3-carbaldehyde (**4**), 3,6-diisobutylpyrazin-2(1H)-one (**5**) [17] and terretrione C (**6**) [18] (Figure 1). We present herein the isolation, structural elucidation and biological activities of these compounds.

## 2. Results and Discussion

### 2.1. Purification of the Compounds **1**–**6**

The mycelia and culture broth of the fungus *Penicillium* sp. CYE-87 were extracted with organic solvents. The combined crude extracts were subjected to partition on reversed-phase C18 silica gel, Sephadex LH-20 and final purification on a C18 reversed-phase semi-preparative HPLC column, which successively led to the isolation of Compounds **1**–**6** (Figure 1).

### 2.2. Structure Elucidation of the Compounds **1**–**6**

**Figure 2:**
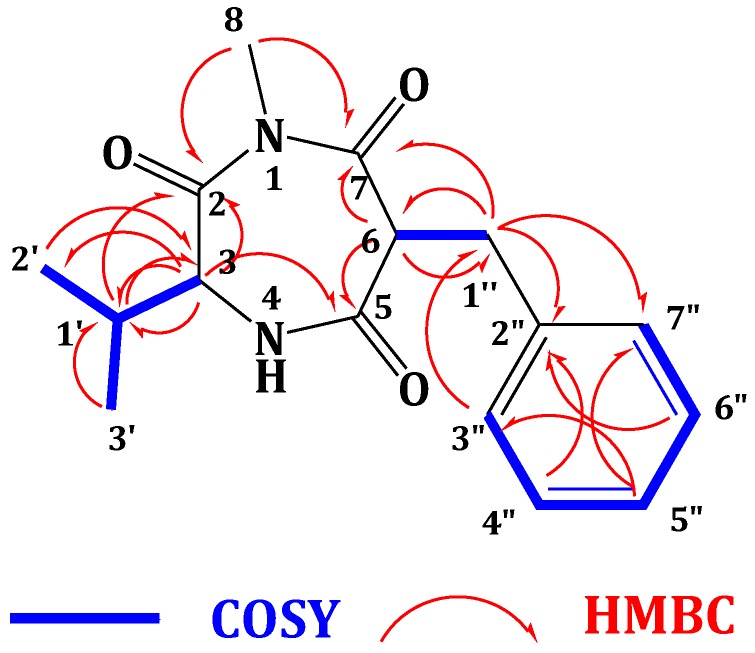
Key COSY and HMBC correlations of **2**.

**Figure 3:**
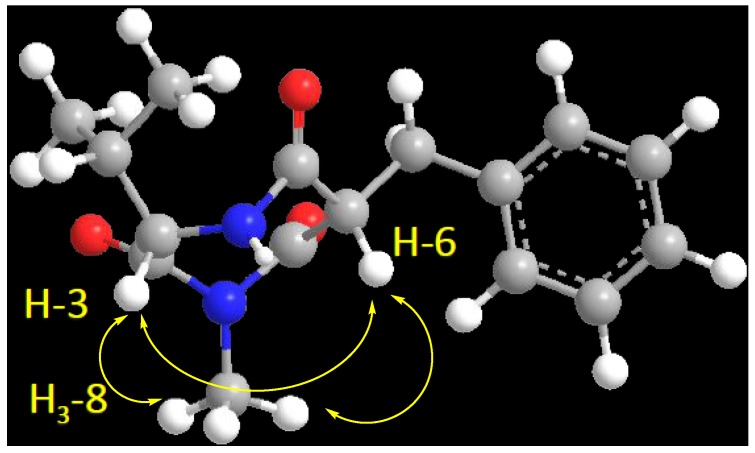
Important H-3 and H-6 NOESY correlation in **2**.

Compound **2** (Figure 1) showed a molecular formula C_16_H_20_N_2_O_3_, as deduced from the HRESIMS pseudomolecular ion peak at *m/z* 289.1555 [M + H]^+^, requiring eight degrees of unsaturation. The ^13^C NMR spectrum revealed signals for 16 carbons. Careful investigation of the ^1^H, ^13^C and 2D NMR spectra supported the presence of a valine residue in **2**. This was evident from the NMR signals at δ_H_/δ_C_ 0.81 (3H, d, *J* = 7.2 Hz, H_3_-2′)/19.8 (C-2′), 0.78 (3H, d, *J* = 7.2 Hz, H_3_-3′)/18.2 (C-3′), 2.07 (1H, m, H-1′)/32.2 (C-1′), 4.18 (1H, d, *J* = 4.8 Hz, H-3)/60.1 (C-3) and δ_C_ 173.0 (C-2). In addition, signals for a 2-benzylmalonamide moiety were observed. The signals at δ_C_ 138.6 (C-2″), δ_H_/δ_C_ 7.23 (2H, m)/130.2 (C-3″,7″), 7.24 (2H, m)/129.4 (C-4″,6″) and 7.14 (1H, m)/127.7 (C-5″) supported the existence of a phenyl group. In addition, the coupled signals at δ_H_ 3.11 (1H, dd, *J* = 13.8, 6.6 Hz, H-1″a) and 2.88 (1H, dd, *J* = 13.8, 8.4 Hz, H-1″b) showed vicinal coupling to the signal at δ_H_ 4.68 (1H, dd, *J* = 8.4, 6.6 Hz, H-6) in the ^1^H,^1^H-COSY spectrum. Furthermore, the two amidic carbonyls (C-5 and C-7) of the 2-benzylmalonamide moiety resonate at δ_C_ 173.2 and 173.1. Finally, a three-proton singlet at δ_H_ 1.90, which correlates with the ^13^C signal at δ_C_ 22.9, was assigned to a methyl group linked to *N*-1. The linkage of the valine residue with the 2-benzylmalonamide moiety through *N*-1 and *N*-4 was supported by the HMBC correlations of H-3/C-5, H-3/C-2, H_3_-8/C-2 (δ_C_ 173.0) and H_3_-8/C-7 (δ_C_ 173.1). The HMBC correlations of H_3_-8 to C-2 and C-7 secured the placement of the *N*-methyl at *N*-1. Additional HMBC correlations within the valine and the 2-benzylmalonamide moieties (Table 1 and Figure 2) confirmed the assignment of all carbon signals and completing the flat structure of **2**. The absolute configuration of the chiral center at C-3 of the valine residue was established by analysis of the degradation product of **2**. A small amount of **2** was hydrolyzed with 6N HCl to give valine residue, which was analyzed by chiral HPLC, and its retention time was compared with the retention times of authentic standards (d- and l-valine). The amino acid in **2** was determined as l-valine. In addition, the relative configuration of C-6 was determined as 6S*, since the NOESY spectrum (Table 1) exhibited a strong correlation between H-3 (δ_H_ 4.18) and H-6 (δ_H_ 4.68). The MM2 ChemBio3D Ultra 14.0 (ChemBioOffice^®^ Ultra 14.0) energy minimized drawing for Compound **2** was also created and supports a significant NOESY correlation between H-3 and H-6 (Figure 3). Therefore, **2** was assigned as (3*S*,6*S*)-6-benzyl-3-isopropyl-1-methyl-1,4-diazepane-2,5,7-trione and named terretrione D. Terretrione D possesses the 1,4-diazepane skeleton, which was previously reported in terretriones A–C. These compounds were reported from the marine fungus *Aspergillus terreus* [18]. It is worth mentioning that terretrione D is 4-demethylterretrione B, and the NMR data of **2** are comparable with those of terretrione B [18]. Compound **2** is reported here for the first time from a natural source, and therefore, it is considered as a new natural product.

The known compounds were identified by extensive study of their spectral data, including HRESIMS, 1D and 2D NMR data, as well as by comparison with the available data in the literature. Thus, the compounds were identified as methyl-2-([2-(1H-indol-3-yl)ethyl]carbamoyl)acetate (**1**) [16], tryptamine (**3**), indole-3-carbaldehyde (**4**), 3,6-diisobutylpyrazin-2(1H)-one (**5**) [17] and terretrione C (**6**) [18]. The amino acid residue of compound **6** was also determined as *N*-methyl-l-isoleucine by HPLC chiral analysis of the hydrolysate of **6** (see Section 3.6). Furthermore, the relative configuration of its C-6 was assigned as 6S* by the NOESY correlation, similar to that described for **2**.

**Table 1:** NMR data of Compound **2** (CD_3_OD, 600 and 150 MHz).

Position	δ_C_, Type ^a^	δ_H_, m (*J* in Hz)	HMBC	NOESY
2	173.0, C ^b^			
3	60.1, CH	4.18, d (4.8)	C-2, C-5, C-1′	H-6, H_3_-8, H-1′
5	173.2, C			
6	56.4, CH	4.68, dd (8.4, 6.6)	C-5, C-7, C-1″	H-3, H_3_-8, H_2_-1″
7	173.1, C ^b^			
8	22.9, CH_3_	1.90, s	C-2, C-7	
1′	32.2, CH	2.07, m	C-2, C-3, C-2′, C-3′	H-3
2′	19.8, CH_3_	0.81, d (7.2)	C-1′, C-3	
3′	18.2, CH_3_	0.78, d (7.2)	C-1′, C-3	
1″	39.0, CH_2_	3.11, dd (13.8, 6.6) 2.88, dd (13.8, 8.4)	C-2″, C-3″, C-6, C-7	H-6
2″	138.6, C			
3″	130.2, CH	7.23, m	C-2″, C-5″	
4″	129.4, CH	7.24, m	C-2″	
5″	127.7, CH	7.14, m	C-3″, C-7″	
6″	129.4, CH	7.24, m	C-2″	
7″	130.2, CH	7.23, m	C-1″, C-5″	

^a^ Multiplicities were deduced by DEPT and HSQC; ^b^ signals may be interchangeable.

### 2.3. Biological Activities of Compounds **1**–**6**

Compounds **1**–**6** were evaluated for their antimigratory activity against the highly metastatic triple-negative human breast cancer cells, MDA-MB-231, their antiproliferation activity against the HeLa cell line, as well as for their antimicrobial activity against three pathogens.

In the wound healing assay to evaluate the migration of MDA-MB-231 cancer cells, Compounds **2** and **6** showed significant antimigratory activity with IC_50_ values of 16.5 and 17.6 μM, respectively, compared to 43.4 μM shown by the positive control *Z*-4-ethylthio-phenylmethylene hydantoin (*S*-Ethyl) (Table 2). All other compounds were weakly active against this cell line. These results clearly suggest that compounds **2** and **6** could be potential candidates for future development of drugs to control metastatic breast cancer. On the other hand, in the antiproliferative assay, compounds **1**–**6** showed weak activity with IC_50_ ≥ 50 µM/mL when tested against HeLa cells. These results of the antimigratory and antiproliferation activities of **1**–**6** are shown in Table 2.

**Table 2:** Antimigatory and antiproliferative activities of **1**–**6**.

Compound	IC_50_ (μM)	
	Antimigratory Activity (MDA-MB-231)	Antiproliferative Activity (HeLa Cells)
**1**	>50	>50
**2**	16.5	>50
**3**	>50	>50
**4**	>50	>50
**5**	>50	>50
**6**	17.6	>50
*S*-Ethyl *	43.4	NT
Paclitaxel *	NT	0.0017

* Positive controls; NT = not tested.

With the exception of Compounds **2** and **6**, all compounds were inactive against all tested pathogens in the antimicrobial screens. Compounds **2** and **6** showed inhibition zones of 17 and 19 mm, respectively, against *C. albicans* in the disc diffusion assay at 100 µg/disc. In addition, these compounds showed MIC with 32 µg/mL against the same strain.

## 3. Experimental Section

### 3.1. General Experimental Procedures

Optical rotations were measured on a JASCO DIP-370 digital polarimeter at 25 °C at the sodium D line (589 nm). The UV spectrum was recorded on a Hitachi 300 spectrometer. 1D and 2D NMR spectra (chemical shifts in ppm, coupling constants in Hz) were recorded on a Bruker Avance DRX 600-MHz spectrometer using CDCl_3_ and CD_3_OD as solvents. NMR spectra were referenced to the residual protonated solvent signals (CHCl_3_: 7.26 ppm for ^1^H and 77.0 ppm for ^13^C; CH_3_OD: 3.30 ppm for ^1^H and 49.0 ppm for ^13^C). Positive ion HRESIMS data were obtained with a Micromass Q-ToF equipped with leucine enkephalin lock spray, using *m*/*z* 556.2771 [M + H]^+^ as a reference mass. For column chromatography, silica gel (Merck, 70–230 mesh ASTM, Darmstadt, Germany) and a Sephadex LH-20 (0.25–0.1 mm, Pharmacia, Piscataway, NJ, USA) were used. Precoated silica gel 60 F-254 plates (Merck, Darmstadt, Germany) were used for TLC. HPLC purifications were performed on a semi-preparative HPLC column (5C_18_-AR-II Cosmosil, 250 × 10 mm, Waters type, Nacalai Tesque, Inc., Kyoto, Japan).

### 3.2. Biological Materials

#### 3.2.1. Collection of the Host Tunicate, *Didemnum* sp.

The marine tunicate *Didemnum* sp. was collected from Suez Canal, Egypt, around a man-made support at a depth of 1–2 m in July 2010. The tunicate was identified by Francoise Monniot at Muséum National d’Histoire Naturelle (MNHN), Paris. A voucher specimen was deposited in the MNHN, Paris, under the Registration Number A2-Did c-476. Another voucher specimen, measuring 2.5–3.0 cm, was deposited in the Red Sea Invertebrates Collection of the Department of Pharmacognosy, Faculty of Pharmacy, Suez Canal University, under Registration Number DY-TOS-1.

#### 3.2.2. Preparation of the Fungal Isolate

In order to ensure fungal isolates to be endophytic when obtained, a surface sterilization of the tunicate was performed. The tunicate sample was disinfected with 5% sodium hypochlorite, followed by 70% ethanol [19], to ensure that epiphytic fungi were destroyed by the washing while associated fungi (if any) were not affected. Approximately 2 cm^3^ of the inner tissue of tunicate material was homogenized using a sterile mortar and pestle containing 10 mL of sterile artificial seawater under aseptic conditions. The resulting homogenate was diluted with sterile seawater at three dilutions (1:10, 1:100 and 1:1000). For fungi cultivation, 100 μL of each dilution were plated in quadruplicate onto four plates of each of the following media; Czapek-Dox yeast agar medium (NaNO_3_ 3.0 g, KCl 0.5 g, K_2_HPO_4_ 0.1 g, MgSO_4_·7H_2_O 0.5 g, FeSO_4_ 0.01 g, sucrose 30.0 g, agar 20.0g, pH 6.7); malt agar medium (malt extract 17.0 g, peptone 3.0 g, agar 20.0 g) and Sabouraud dextrose agar medium. All media were amended with 2% NaCl and 0.25% chloramphenicol as the antibacterial agent to prevent bacterial growth and to enrich fungal growth. Plates were wrapped in parafilm, incubated at 28 °C for 1–3 weeks, until the morphology of fungi could be distinguished. Many purification steps were done until pure fungal isolates were obtained.

#### 3.2.3. Extraction of Genome DNA from Cultured Fungal Isolate CYE-87

The fungal isolate was subcultured in corresponding broth at 28 °C for 2–5 days. The mycelia were harvested separately by using vacuum filtration and dried with two layers of paper towel. The resulting mycelial mat was ground into powder with liquid nitrogen. The fungal DNA was extracted using the QIAamp DNA Mini Kit (Qiagen, Valencia, CA, USA) according to the manufacturer’s instructions. 

#### 3.2.4. Amplification of Fungal ITS-rDNA Fragments of Isolate CYE-87

The genomic DNA of the fungal strain was used as the template to amplify fungal Internal Transcribed Spacer-rDNA (ITS-rDNA) fragments using the primers ITS1 (5′-TCCGTAGGTGAACCTGCG-3′) and ITS4 (5′-TCCTCCGCTTATTGATATGC-3′) [20], which were synthesized by the University of Utah DNA/peptide synthesis core facility. The reaction mixture for PCR amplification contained 5 μL of 10× reaction buffer with 15 mM MgCl_2_ (Invitrogen), 2 μL of 2.5 mM dNTPs, 0.5 μL of 10 µM of each primer, 4 μL of fungal DNA, 0.3 μL of Taq DNA polymerase (5 U·μL^−1^, Invitrogen) and 39.7 μL of H_2_O. PCR conditions included an initial denaturation at 94 °C for 4 min followed by 30 cycles of denaturation at 94 °C for 50 s, annealing at 51 °C for 50 s and elongation at 68 °C for 1 min, with a final elongation at 68 °C for 10 min. PCR products were purified using the Agarose Gel DNA Purification Kit (Qiagen, Valencia, CA, USA) and sequenced at the University of Utah DNA sequencing facility.

#### 3.2.5. Sequence Fungal ITS-rDNA Regions of Isolate CYE-87

For preliminary identification, the sequence of fungal ITS-rDNA regions obtained from the marine tunicate *Didemnum* sp. were compared with related sequences at the National Center for Biotechnology Information (NCBI) [21]. Fungal ITS-rDNA sequences acquired in this study were edited and aligned with the best n-BLAST hits from GenBank in the Clustal X (Version 1.83) program [22] and further manually adjusted using BioEdit software [23]. The program MEGA 5 [24] was applied to calculate the base composition of the fungal sequence. 

#### 3.2.6. Characterization of the Fungal Isolate CYE-87 

The sequence analysis of the fungal strain CYE-87 showed 99% sequence identity with *Penicillium* sp. GP2 (NCBI Accession Number DQ875010.1). A voucher specimen under the number CYE-87 was deposited in the Faculty of Pharmacy Culture Collection at King Abdulaziz University, Jeddah, Kingdom of Saudi Arabia.

### 3.3. Culture Condition and Extraction

The colonies were directly inoculated into 1,000-mL Erlenmeyer flasks, each containing 250-mL Czapek yeast media (NaNO_3_ 3.0 g, K_2_HPO_4_ 1.0 g, MgSO_4_·7H_2_O 0.5 g, KCl 0.5 g, FeSO_4_ 0.01 g, yeast extract 5.0 g, sucrose 30.0 g and NaCl 20.0 g, dissolved in distilled water 1,000 mL). The cultures were incubated on a shaking bed at 120 rpm at 26 °C. After 14 d of cultivation, 20-L cultures were filtered through cheesecloth to separate the culture broth and mycelia. The former was successively extracted with EtOAc to afford the crude extract (1.5 g). The mycelia were also extracted by MeOH with ultrasound to achieve mycelial extract (2.2 g).

### 3.4. Isolation and Purification of Compounds **1**–**6**

The broth and mycelia extracts showing similar TLC patterns were combined together, and a fraction of the combined extracts (1.0 g) was subjected to VLC chromatography on RP C18 silica using water/MeOH gradients to give five fractions (A–E). Fraction C (180 mg), which was eluted with 30% MeOH in H_2_O, was subjected to a Sephadex LH-20 column eluted with MeOH to give four main subfractions (C1–C4). Fraction C2 (70 mg) was subjected to HPLC purification on a C18 semipreparative column using 40% ACN in H_2_O to afford Compounds **2** (3.4 mg) and **6** (2.1 mg). Similarly, Subfraction C3 (50 mg) was subjected to HPLC purification on a semipreparative column using 40% ACN in H_2_O to afford Compounds **1** (2.9 mg), **3** (15.5 mg) and **4** (9.5 mg). Finally, Fraction C4 (25 mg) was subjected to a semipreparative HPLC column using 25% ACN in H_2_O to afford Compounds **5** (3.3 mg). 

**Terretrione D** (**2**): White amorphous powder; [α]_D_ −65.7 (*c* 1.5, MeOH); UV (MeOH) λ_max_ (log ε) 314 (2.65) nm; IR ν_max_ (film) 3367, 1682, 1630, 1540, 1455, 1288, 1094, 702 cm^−1^; NMR data, see Table 1; HRESIMS *m*/*z* 289.1555 (calcd. for C_16_H_21_N_2_O_3_, [M + H]^+^, 289.1552). 

### 3.5. Absolute Stereochemistry of the Amino Acids in **2** and **6**

Compounds **2** and **6** (each 0.5 mg) were hydrolysed separately in 6 N HCl at 105 °C for 16 h, then dried under a stream of N_2_ and further dried under vacuum. The residue was reconstituted with 350 μL of H_2_O prior to chiral HPLC analysis. For column Phenomenex Chirex 3126 (D), 4.6 × 250 mm; UV 254-nm detector, Mobile Phase 1: 100% 2 mM CuSO_4_ in H_2_O, flow rate 0.7 mL/min; Mobile Phase 2: 2 mM CuSO_4_ in MeCN/H_2_O (15:85), flow rate 0.8 mL/min. Mobile Phase 1 elution times (*t*_R_, min) of authentic standard: l-valine (38.4), d-valine (68.5). Mobile Phase 2 elution times (*t*_R_, min) of authentic standard: *N*-methyl-l-isoleucine (18.8), *N*-methyl-d-isoleucine (28.2). The hydrolysate was chromatographed alone and co-injected with standards to confirm assignments as l-valine (in Compound **2** hydrolysate) and *N*-methyl-l-isoleucine (in Compound **6** hydrolysate).

### 3.6. Biological Activities of the Compounds

#### 3.6.1. Evaluation of the Antimigratory of **1**–**6** Using the Wound Healing Assay

The wound healing assay is a simple method for evaluating directional cell migration *in vitro*. All compounds were tested for the ability to inhibit the migration of highly metastatic triple-negative human breast cancer cells MDA-MB-231 using the wound-healing assay model. A vehicle (DMSO) and *Z*-4-ethylthio-phenylmethylene hydantoin (*S*-Ethyl) were used as negative and positive controls. The assay was conducted as described previously [25]. Briefly, cells were plated on sterile 24-well plates and allowed to form a confluent monolayer per well (>90% confluence) overnight. Wounds were then inflicted in each cell monolayer using a sterile 200 μL pipette tip. The medium was removed, and cells were washed twice with PBS and once with fresh RPMI medium. Test compounds at the desired concentrations were prepared in fresh medium (0.0% or 0.5% FBS) and were added to wells in triplicate. The incubation was carried out for 24 h, after which the medium was removed and cells were washed, fixed and stained using Diff-Quick™ staining (Dade Behring Diagnostics, Aguada, Puerto Rico). Cells that migrated across the inflicted wound were counted under the microscope in at least five randomly selected fields (magnification: 400×). The results are shown in Table 2.

#### 3.6.2. Evaluation of Antiproliferative and Cytotoxic Activities 

The effect of the Compounds **1**–**6** on HeLa cell proliferation and cytotoxicity were evaluated using the sulforhodamine B (SRB) assay [26,27,28]. HeLa cells were grown in Basal Medium Eagle (BME) containing Earle’s salts, 10% FBS and 50 μg/mL gentamycin sulfate. Cells were plated at a density of 2500 cells per well in a 96-well plate and allowed to adhere and grow for 24 h before compounds were added. The compounds were solubilized in DMSO and added to a final DMSO concentration of 1% in both test wells and vehicle controls. The cells were incubated with compounds or vehicle for an additional 48 h. The IC_50_, the concentrations required to cause a 50% inhibition of cell proliferation, were calculated from the log dose response curves. The values represent the average of 3–4 independent experiments, each conducted in triplicate ± SEM. Cytotoxicity was determined by a cell density lower than that measured at the time of drug addition. Paclitaxel was used as a positive control. The results are shown in Table 2.

#### 3.6.3. Evaluation of the Antimicrobial Activity of Compounds **1**–**6**

##### 3.6.3.1. Determination of the Antimicrobial Activities Using the Disc Diffusion Assay

The *in vitro* antimicrobial activity was evaluated using the disc diffusion method, as previously described [29]. Varieties of test microorganisms were used, including a Gram-positive bacterium (*Staphylococcus aureus* ATCC 25923), a Gram-negative bacterium (*Escherichia coli* ATCC 25922) and yeast (*Candida albicans* ATCC 14053). The adjusted inoculum of each microorganism, equivalent to a turbidity of 0.5 McFarland standards, was streaked separately using sterile swabs over the surface of Muller-Hinton agar plates. Sterile filter paper discs (6 mm diameter) were impregnated with 100 μg of each compound and applied to the inoculated plates. The plates were incubated at 37 °C for 24 h. Solvent control discs were used to determine any solvent effect. Gentamycin and ciprofloxacin were used as antibacterial standards, while nystatin was used as an antifungal standard. The activity of each compound was determined by measuring the diameter of the inhibition zone in mm. The technique was performed in duplicate, and the mean diameter of each inhibition zone was recorded. 

##### 3.6.3.2. Determination of the MIC Values against *C. albicans*

Due to the activity of the compounds against *C. albicans* in the disc diffusion assay, the minimum inhibitory concentration (MIC) values of the compounds were determined against this strain. The MIC values of Compounds **1**–**6** were determined against *C. albicans* following the National Center for Clinical Laboratory Standards (NCCLS) methods [30,31]. Briefly, compounds (dissolved in DMSO) were serially diluted in 20% DMSO/saline and transferred (10 μL) in duplicate to 96-well flat-bottom microplates. The fungal strain was grown aerobically at 0 °C in Sabarouad Dextrose Agar (SDA) for 16–20 h. A set of different concentrations of Compounds **1**–**6** prepared in RPMI 1640 medium were next inoculated with the microorganism and incubated for 46 h at 35 °C. The MIC (μg/mL) was defined as the lowest concentration of the compound that inhibited the visible growth. The MIC values were evaluated in triplicate for each compound. Fluconazole was used as the positive control. 

## 4. Conclusions 

In conclusion, the investigation of the marine-derived fungus *Penicillium* sp. CYE-87 yielded a new compound with the 1,4-diazepane moiety with the name terretrione D (**2**) together with five known compounds, including methyl-2-([2-(1H-indol-3-yl)ethyl]carbamoyl)acetate (**1**), tryptamine (**3**), indole-3-carbaldehyde (**4**), 3,6-diisobutylpyrazin-2(1H)-one (**5**) and terretrione C (**6**). The structures of **1**–**6** were determined by interpretation of their spectral data, including HRESIMS and 1D and 2D NMR. Compounds **2** and **6** showed significant antimigratory activity against the highly metastatic triple-negative human breast cancer cells MDA-MB-231 with IC_50_ values of 16.5 and 17.6 μM, respectively. On the other hand, Compounds **2** and **6** showed moderate activity against *C. albicans* with a MIC value of 32 µg/mL.
